# Both Granulocytic and Non-Granulocytic Blood Cells Are Affected in Patients with Severe Congenital Neutropenia and Their Non-Neutropenic Family Members: An Evaluation of Morphology, Function, and Cell Death

**DOI:** 10.4274/tjh.2017.0160

**Published:** 2018-11-13

**Authors:** Lale Olcay, Şule Ünal, Hüseyin Onay, Esra Erdemli, Ayşenur Öztürk, Deniz Billur, Ayşe Metin, Hamza Okur, Yıldız Yıldırmak, Yahya Büyükaşık, Aydan İkincioğulları, Mesude Falay, Gülsüm Özet, Sevgi Yetgin

**Affiliations:** 1 Ankara Oncology Training and Research Hospital, Clinic of Pediatric Hematology, Ankara, Turkey; 2 Hacettepe University Faculty of Medicine, İhsan Doğramacı Children’s Hospital, Clinic of Pediatric Hematology, Ankara, Turkey; 3 Ege University Faculty of Medicine, Department of Medical Genetics, İzmir, Turkey; 4 Ankara University Faculty of Medicine, Department of Histology Embryology, Ankara, Turkey; 5 Ankara University Faculty of Medicine, Department of Pediatric Molecular Genetics, Ankara, Turkey; 6 Ankara Children’s Hematology Oncology Training and Research Hospital, Clinic of Pediatric Immunology, Ankara, Turkey; 7 Şişli Etfal Children’s Training and Research Hospital, Clinic of Pediatric Hematology, İstanbul, Turkey; 8 Hacettepe University Faculty of Medicine, Department of Internal Medicine, Unit of Hematology, Ankara, Turkey; 9 Ankara University Faculty of Medicine, Department of Pediatric Immunology and Allergy and Pediatric Molecular Genetics, Ankara, Turkey; 10 Ankara Numune Training and Research Hospital, Clinic of Hematology, Ankara, Turkey; 11 Yıldırım Beyazıt University Faculty of Medicine, Department of Internal Medicine, Clinic of Hematology, Ankara, Turkey

**Keywords:** Severe congenital neutropenia, Monocytes, Lymphocytes, NK cells, Thrombocytes, Phagocytes, Apoptosis, Senescence, Parents, Family

## Abstract

**Objective:**

To examine granulocytic and non-granulocytic cells in children with severe congenital neutropenia (SCN) and their non-neutropenic parents.

**Materials and Methods:**

Fifteen patients with SCN and 21 non-neutropenic parents were evaluated for a) CD95, CD95 ligand, annexin V, propidium iodide, cell cycle, and lymphocyte subsets by flow cytometry; b) rapid cell senescence (of leukocytes) by senescence-associated β-galactosidase stain; c) aggregation tests by aggregometer; d) in vitro bleeding time by PFA-100 instrument; e) mepacrine-labeled dense granule number of thrombocytes by fluorescence microscope; and f) hematomorphology by light and electron microscope. *HAX1, ELANE, G6PC3, CSF3R*, and *JAGN1* mutations associated with SCN were studied in patients and several parents.

**Results:**

Significant increase in apoptosis and secondary necrosis in monocytes, lymphocytes, and granulocytes of the patients and parents was detected, irrespective of the mutation type. CD95 and CD95 ligand results implied that apoptosis was non-CD95-mediated. Leukocytes of 25%, 12.5%, and 0% of patients, parents, and controls showed rapid cell senescence. The cell cycle analysis testable in four cases showed G1 arrest and apoptosis in lymphocytes of three. The patients had *HAX1* (n=6), *ELANE* (n=2), G6PC3 (n=2), and unidentified (n=5) mutations. The CD3, CD4, and NK lymphocytes were below normal levels in 16.6%, 8.3%, and 36.4% of the patients and in 0%, 0%, and 15.4% of the parents (controls: 0%, 0%, 5.6%). The thrombocytes aggregated at low rates, dense granule number/thrombocyte ratio was low, and in vitro bleeding time was prolonged in 37.5%-66.6% of patients and 33.3%-63.2% of parents (vs. 0% in controls). Under electron and/or light microscope, the neutrophils, monocytes, lymphocytes, and thrombocytes in the peripheral blood of both patients and parents were dysplastic and the bone marrow of patients revealed increased phagocytic activity, dysmegakaryopoiesis, and necrotic and apoptotic cells. Ultrastructurally, thrombocyte adhesion, aggregation, and release were inadequate.

**Conclusion:**

In cases of SCN, patients’ pluripotent hematopoietic stem cells and their non-neutropenic parents are both affected irrespective of the genetic defect.

## Abstract (tr)

**Amaç:**

Ciddi konjenital nötropenisi (CKN) olan hastalar ve nötropenik olmayan ebeveynlerindeki granülositik ve granülositik olmayan kan hücrelerini incelemektir.

**Gereç ve Yöntemler:**

CKN’si olan 15 çocuk ve nötropenik olmayan 21 ebeveynin lenfosit, granülosit ve monositlerinde CD95, CD95 ligand, annexin V, hücre siklusu (periferik lenfositler, granülosiler +/- monositlerde) ve lenfosit alt grupları akım sitometri ile, b) hızlı hücre yaşlanması (lökositlerde) yaşlanma-ilişkili β-galaktozidaz boyası SA-β-galaktosidaz boyası ile, c) agregasyon testleri agregometre ile, d) in vitro kanama zamanı, PFA-100 aleti ile, e) trombositlerde mepakrin işaretli kaba granül sayısı floresan mikroskopu ile, f) hematomorfoloji ışık ve elektron mikroskopu ile değerlendirildi. Hastalarda ve bazı ebeveynlerde CKN ile ilişkili olarak *HAX1, ELANE, G6PC3, CSF3R, JAGN1* mutasyonları çalışıldı.

**Bulgular:**

Akım sitometri ile, hasta ve ebeveynlerinin monosit, lenfosit ve granülositlerinde apoptoz ve sekonder nekrozda belirgin artış olduğu ve bunun konjenital nötropeni mutasyonunun cinsi ile ilişkili olmadığı gösterildi. CD95 ve CD95 ligand sonuçları, apoptozun CD95 yolu ile olmadığını gösteriyordu. Hasta, ebeveyn ve kontrol olgularının lökositlerinin %25, %12,5 ve %0’ı SA-β-gal boyası ile boyandı. Dört olguda yapılabilen hücre siklusu analizinde üç olgunun lenfositlerinde G1 arresti ve apoptoz görüldü. Hastalarda *HAX1* (n=6); *ELANE* (n=2); G6PC3 (n=2) ve belirlenemeyen (n=5) mutasyonlar saptandı. CD3, CD4 ve NK lenfositleri sırasıyla hastaların %16,6; %8,3; %36,4’ünde, ebeveynlerin %0, %0, %15,4’ünde, kontrolün %0, %0, %5,6’sında yaşa göre normal aralığın altında idi. Hasta ve ebeveynlerin trombositleri düşük oranda agrege oluyordu (olguların sırasıyla %66,6 ve %63,2’sinde, kontrolün %0’ında), kaba granül sayısı/trombosit oranı düşük (hasta, ebeveyn ve kontrolün %50, %35 ve %0’ında); in vitro kanama zamanı uzun (farklı kartuşlarla olguların %37,5 ve %33,3’ünde ve ebeveynlerin %18,8 ve %12,5’inde) idi. Işık ve elektron mikroskopta hasta ve ebeveynlerin periferik kanlarındaki nötrofil, monosit, lenfosit ve trombositleri displastik idi; hastaların kemik iliğinde fagosit aktivitesinde artış, dismegakaryopoez, nekrotik ve apoptotik hücreler bulunuyordu. İnce yapısal olarak trombositlerde adezyon, agregasyon, salınım yetersiz idi.

**Sonuç:**

CKN’de, pluripotent hematopoietik kök hücreler ve nötropenik olmayan ebeveynleri genetik bozukluktan bağımsız olarak etkilenirler.

## Introduction

Severe congenital neutropenia (SCN) is a heterogeneous bone marrow failure syndrome characterized by recurrent infections, low absolute neutrophil count (<0.5x10^9^/L), and maturation arrest at the promyelocyte/myelocyte stage of myelopoiesis in the vast majority of cases and it is due to various genetic defects [1,2,3]. Regular variations [4], giving rise to transient elevations of neutrophil counts to even >1.5x10^9^/L with ‘intermittent maturation arrest’ [5], can be encountered.

Apoptosis in neutrophilic precursors plays a major role in the pathogenesis of SCN [1,2,6]. Reports regarding lymphocyte apoptosis in addition to granulocyte apoptosis have been restricted to a few cases [7,8], and apoptosis in monocytes has not been studied. Reports pertaining to non-granulocytic blood cell lines in SCN and patients’ non-neutropenic family members are also too limited [3,7,8,9,10] to make a general characterization of the phenotype of SCN cases with heterogeneous genetic backgrounds. 

We have hypothesized that, in SCN, development of all cell lines other than the granulocytic lineage is also impaired and patients’ non-neutropenic parents also carry some hematologic abnormalities. Our specific aim in this study is to examine the lymphocytes, monocytes, and granulocytes of patients with SCN and their family members in terms of morphology and cell death parameters [apoptosis and rapid cell senescence (RCS)] and additionally to evaluate thrombocyte morphology and functions and percentage of lymphocyte subsets.

## Materials and Methods

### Study Participants

Severe congenital neutropenia was defined as persistent neutropenia (neutrophil counts of <0.5x10^9^/L) confirmed from two samples a week for 6 weeks and the onset of neutropenia or infections early in life and deficiency in late maturation stages of neutrophils in bone marrow (mature neutrophils being <10%: central neutropenia) [4]. However, subjects whose neutrophils showed some spontaneous variations between <0.5x10^9^/L and 1.5x10^9^/L were not excluded [4]. Those with syndromic neutropenia were excluded. 

Fifteen children with SCN [age: 9.35±4.54 years; range: 1.5-22; 8 female (F), 7 male (M)] and 21 non-neutropenic family members (10 mothers, 11 fathers; age: 35.14±8.92 years; range: 23-55) were included in the study. A 22-year-old female was included since she had been followed in Pediatrics for 8 years.

Patients were prescribed G-CSF (5-25 µg/kg/day), 2-7 times weekly; however, many patients received therapy irregularly for economic and social reasons. Blood was drawn during periods in which patients had stopped therapy and patients and family members had not consumed any other drugs for at least 10 days prior to samples being taken to eliminate drug effects on thrombocyte aggregation [11]. 

For lymphocyte subsets, death parameters (CD95, CD95 ligand, annexin V), dysmorphism, thrombocyte aggregation tests, mepacrine labeling, and in vitro bleeding time, 18, 10, 9, 5, 5, and 9 healthy volunteers were evaluated, respectively. For evaluation of lymphocyte subsets, age-matched normal ranges for healthy Turkish children [12] and our laboratory for adults were used.

### Flow Cytometric Evaluation

**Figure 1:**
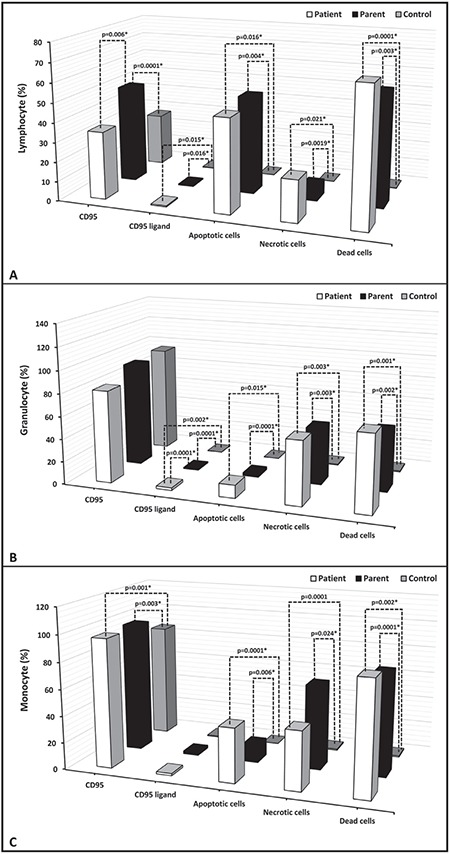
Percentage of CD95, CD95 ligand, annexin (showing apoptotic cells), propidium iodide (PI), or 7-aminoactinomycin D (7-AAD) (showing necrotic cells) and overall dead cells (apoptotic + necrotic cells) in lymphocyte, monocyte, and granulocyte gates.

Peripheral blood was prepared for flow cytometric analysis as reported previously [8,13,14] by direct immunofluorescence (FAC Scan, Becton Dickinson CA and Beckman Coulter, USA). By combining CD45 and CD14 with the forward and right-angle light scatter parameters of blood cells, the lymphocytes, granulocytes, and monocytes were gated (Figure 1A). CD95, CD95 ligand, and annexin V were evaluated in each gate; the CD3, CD4, CD8, and NK cells were evaluated in the lymphocyte gates [13,14]. Kits from Biosciences (USA) (for CD95 and CD95 ligand) and Pharmingen (USA) [for annexin V, propidium iodide (PI), 7-aminoactinomycin D (7-AAD), and CD3, CD4, CD8, and NK cells] were used. Per sample, 10,000 cells were counted. The cell cycles of the lymphocytes and granulocytes were evaluated by the PI florescence histogram method [13].

### Rapid Cell Senescence

The leukocytes were stained for senescence-associated β-galactosidase (SA-β-gal kit, Sigma Co., Germany) as per the manufacturer’s protocols [15].

### Mutation Analysis

Mutation analyses of* HAX1, ELANE, CSF3R, G6PC3, *and* JAGN1 *genes were performed by standard techniques (Supplemental Materials and Methods and Supplemental Table 1).

### Evaluation of Cellular Morphology

The peripheral blood cells were evaluated by light (Nikon E400) and transmission electron microscopy (TEM) (LEO 906E) for apoptosis and dysplasia [8,10,14,16,17], in a blinded fashion (Supplemental Materials and Methods). The bone marrow aspiration smears taken at admission were also evaluated under light microscope. Bone marrow aspiration of the parents could not be performed.

### Evaluation of the Thrombocytes of the Patients and the Parents

In vitro bleeding time was measured with a PFA-100 instrument (Dade Behring Marburg GmbH, Marburg, Germany) [18] and turbidimetric aggregation tests were measured with a Chrono-Log 560 Ca aggregometer (Chrono-Log Corporation, Havertown, PA, USA) [18]. 

Dense granules were stained with mepacrine (1 µM, Sigma, St. Louis, MO, USA) [19,20] and thrombocytes were prepared for electron microscopic visualization of aggregation [19], as described previously (Supplemental Materials and Methods).

### Statistics Analysis

We used SPSS 15.0 (SPSS Inc., Chicago, IL, USA) to evaluate the data we obtained. A normality test was performed to determine if the data were distributed in a normal fashion (Supplemental Materials and Methods).

## Results

### History and Physical Examination

In our cohort, there were three pairs of siblings and one pair of cousins, one having coexistent amyloidosis and hypercholesterolemia and the other having hemoglobin C. Their vaccines were administered on time without any complications. Parents of 14 patients were 1^st^ (n=10) or 2^nd^ (n=4) degree relatives. The patients had gingival hypertrophy, aphthous stomatitis, decayed teeth, and tooth loss by 26.6%, 20%, 20%, and 13.3%, respectively. None had any physical malformation. Several patients had monocytosis and thrombocytosis [21,22]. The immunoglobulin (Ig) A, G, and M levels of patient AG and the IgG of patient ZG were higher than normal, while the levels of all the other patients were normal. Four out of 15 SCN patients (26.6%) and 5 of 21 parents (23.8%; 3 mothers, 2 fathers) had frequent nasal bleeding and easy bruising with/without menorrhagia. Investigations of immunoglobulin levels, which could be performed for ten parents, revealed normal results. The other characteristics of the patients and parents are presented in Table 1 and Supplemental Table 2.

### Flow Cytometric Evaluation

Percentage of apoptotic cells, necrotic cells, and dead cells (apoptotic + necrotic) in the lymphocyte, granulocyte, and monocyte gates of both the patients and the parents were higher than those of the healthy controls, while they were similar among the patients and parents (Figure 1). CD95 and CD95 ligand results were inconsistent with each other, implying that apoptosis was non-CD95-mediated (Table 2; Figure 1). 

The CD3, CD4, and NK cells were below the age-matched normal ranges in 16.6%, 8.3%, and 36.4% of the patients and 0%, 0%, and 15.4% of the parents versus 0%, 0%, and 5.6% of the controls. On the other hand, CD3 and CD8 cells were found to be above the age-matched normal ranges in 16.6% and 27.3% of the patients and in 0% and 7.7% of the parents versus 0% and 16.7% of the controls (Supplemental Table 3).

**Figure 3:**
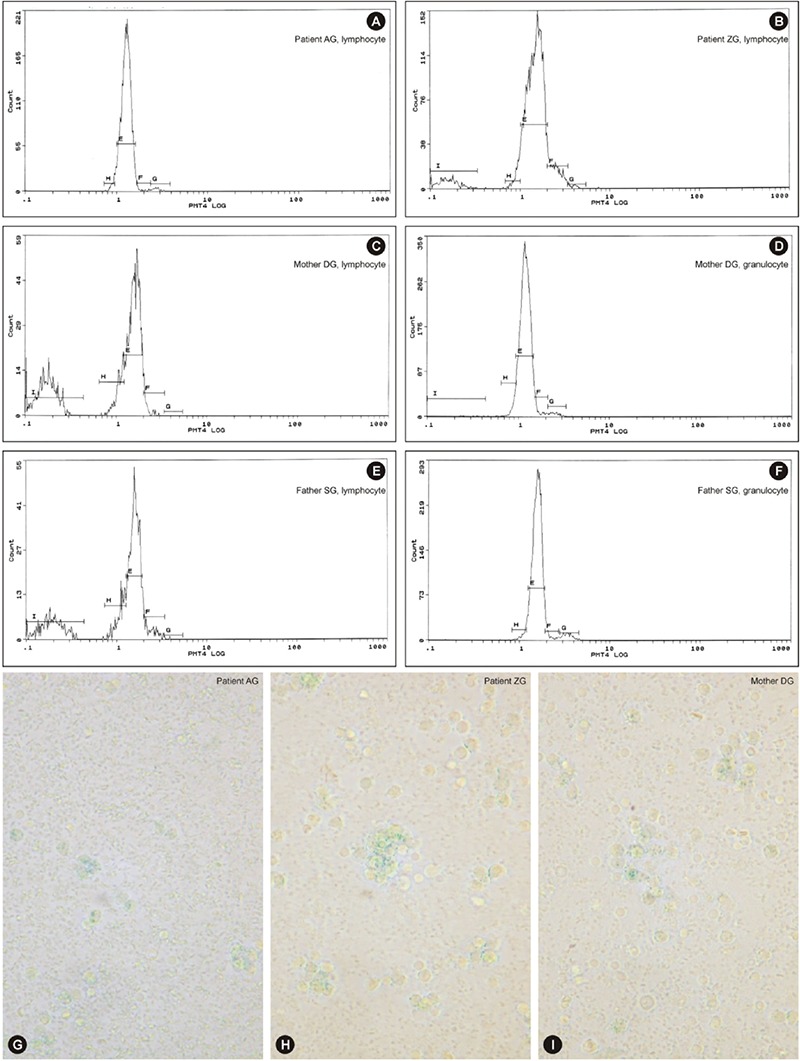
Cell cycle patterns of two sibling patients, their mother, and their father: A) normal cell cycle (patient AG, lymphocyte; sibling of ZG); B) G1 arrest and pre-G1 peak showing apoptosis (patient ZG; sibling of AG); C) G1 arrest and apoptosis (mother DG, lymphocyte); D) normal cell cycle (mother DG, granulocyte); E) G1 arrest and apoptosis (father SG, lymphocyte); F) normal cell cycle (father SG, granulocyte) (the patients’ granulocytes could not be evaluated due to granulocytopenia). G-H-I: The leukocytes of two sibling patients and their mother stained by SA-β-gal, as blue granules, from peripheral blood culture (400^x^). The leukocytes of patient AG (G), patient ZG (H), and their mother DG (I). These patients were members of a family evaluated for cell cycles.

G1 arrest and apoptosis were established in one patient’s lymphocytes (ZG) and those of her parents while the cell cycle of lymphocytes in the sibling of ZG (patient AG) was normal. The cell cycles of the parents’ granulocytes were normal (Figures 3A-3F).

### Rapid Cell Senescence

Eight patients and eight parents were evaluated. The leukocytes of only 2 patients (siblings AG and ZG) and their mother were stained with SA-β-gal by 88%, 76%, and 94%, respectively (Figures 3G, 3H, 3I). These patients were members of a family and were evaluated for cell cycles at the same time (Figures 3A-3F).

## Cell Morphology

### Neutrophils

The incidences of bizarre nuclei (34.0±17.4% vs. 15.2±4.7%, p=0.015), pseudo Pelger-Huet (PPH) and PPH-like cells (15.7±9.3 vs. 4.1±3.3, p=0.003), striking chromatin clumping (38.1±27.7% vs. 11.0±9.7%, p=0.036), macropolycyte percentage (diameter ≥15 µm) (38.71±27.46% vs. 6.44±6.00%, p=0.0001) of neutrophils, and neutrophil diameter (13.46±1.76 vs. 9.79±1.73, p=0.0001) were significantly higher in the neutrophils of the parents than those of the control group by light microscope.

The few neutrophils of the patients in their peripheral blood and bone marrow also revealed the same abnormalities, but no scoring could be done due to the low number.

**Figure 4:**
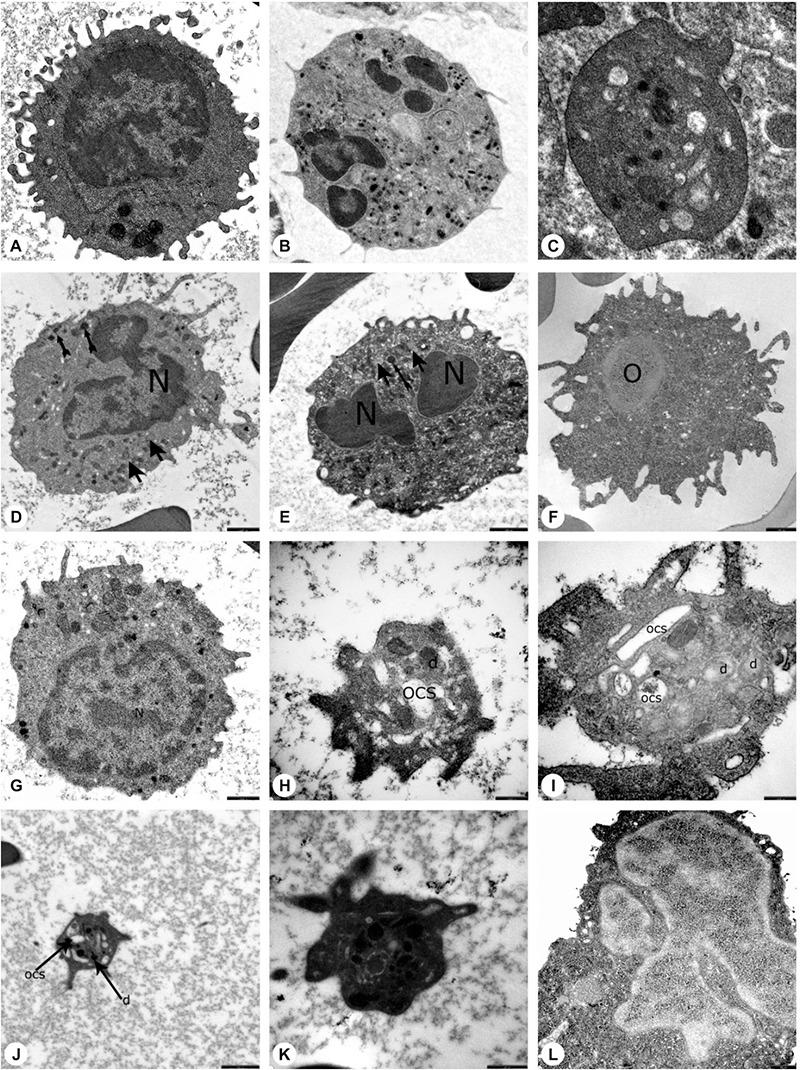
Electron microscopic images of the blood cells of the patients. A) Normal lymphocyte (16700^x^), B) normal neutrophil (3597^x^), C) normal thrombocyte (10000^x^), D) patient EC (with *HAX1* mutation) (12930^x^), E) patient NBÖ (with *ELANE* mutation) (12930^x^, F) patient OSK (with *G6PC3* mutation) (10000^x^), G) patient EC (with *HAX1* mutation) (16700^x^), H) patient EC (with *HAX1* mutation) (35970^x^), I) patient MeK (with *G6PC3* mutation) (46460^x^), J) patient MNY (with *HAX1* mutation) (12930^x^), K) patient NBÖ (with *ELANE* mutation) (27800^x^), L) patient MeK (with *G6PC3* mutation) (14000^x^) (N: nucleus; thick arrow: primary granule; thin arrow: secondary granule; OCS: open canalicular system; d: dense granule; *: segment of non-apoptotic nucleus; arrow head: fusion of granules; O: autophagosome). Decreased number of secondary granules in the neutrophils (Figures 4D, 4E), which were abnormal in shape (4L). Primary granules that were irregular in shape (Figures 4E, 4L) or large (Figure 4D) and had a tendency to combine and condense (Figure 4E). Chromatin clumping in nuclei (Figure 4D) and apoptosis (Figure 4E). An active lymphocyte (Figure 4G). Abnormal giant mononuclear cell with abundant mitochondria and autophagic vacuole (Figure 4F). Dense granules in platelets, which were large and giant (Figure 4H), reduced in number (Figure 4H), in different shapes and dimensions (Figures 4H, 4J, 4K) with varying components (Figure 4I). Enlarged open canalicular system due to unevacuated ingredients (Figures 4H, 4I, 4J, 4K).

**Figure 5:**
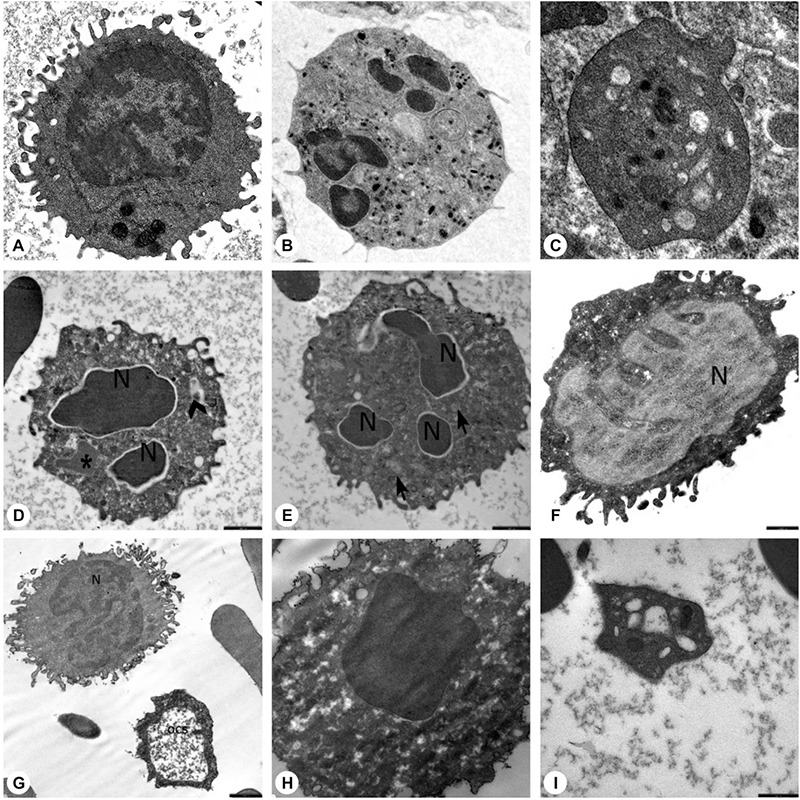
Electron microscopic images of the blood cells of the parents. A) Normal lymphocyte (16700^x^), B) normal neutrophil (3597^x^), C) normal thrombocyte (10000^x^), D) mother of AO (with unidentified mutation) (SO) (12930^x^), E) mother of AO (with unidentified mutation) (SO) (12930^x^), F) father of MeK and OSK (with *G6PC3* mutation) (MK) (16700^x^), G) father of MeK and OSK (with *G6PC3* mutation) (MK) (10000^x^), H) father of MeK and OSK (with *G6PC3* mutation) (MK) (21560^x^), I) father of MNY and AY (with *HAX1* mutation) (AhY) (27000^x^) (N: nucleus; thick arrow: primary granule; thin arrow: secondary granule; OCS: open canalicular system; d: dense granule; *: segment of non-apoptotic nucleus; arrow head: fusion of granules; O: autophagosome). Decreased number of secondary granules in the neutrophils (Figures 5D, 5E). Primary granules in irregular shape (Figure 5D). Chromatin clumping in nuclei (Figure 5D) and apoptosis (Figures 5D, 5E, 5H). Abnormal lymphocytes (Figures 5F, 5G). Dense granules in platelets, which were large and giant (Figure 5I). Open canalicular system enlarged due to unevacuated ingredients (Figures 5G, 5H, 5I).

Ultrastructural study of the patients and parents revealed that secondary granules of neutrophils were low in number, primary granules were heterogeneous in shape and size, and chromatin clumping and apoptosis were striking (Figures 4D, 4E, 4L, 5D, 5E, and 5H).

### Lymphocytes

The peripheral lymphocytes of both patients and parents revealed few lymphocytes with tiny cytoplasmic protrusions. Ultrastructural study of peripheral blood of patients and parents showed that the lymphocytes were abnormal or active (Figures 4G, 5F, and 5G).

### Monocytes, Macrophages, Histiocytes, and Other Phagocytes

The peripheral blood of patients and parents revealed monocytes with features of apoptosis, abnormal nucleus, necrosis, or pseudopod formation under light microscope and abnormal mononuclear cells under TEM with or without features of apoptosis (Figures 4F and 5H).

The bone marrow examination of the patients revealed many monocytes, macrophages, histiocytes, neutrophils, bands, eosinophils, and eosinophil myelocytes undergoing phagocytosis. Sea blue-like and Gaucher-like histiocytes in the bone marrow of the patients were striking. The phagocytosed cells were lymphocytes, erythroblasts, or apoptotic cells. Necrosis of cells that had phagocytosed other cells was also evident (Figures 6A, 6B, 6C).

### Megakaryopoiesis

Megakaryocytes with asynchrony in nucleo-cytoplasmic maturation or those undergoing emperipolesis or transformed/transforming to naked megakaryocyte nuclei were striking. Additionally, naked megakaryocyte cytoplasm just after completing thrombocyte release, many megakaryoblasts, and necrotic, apoptotic, or dysplastic megakaryocytes were also seen in the bone marrow examinations of the patients (Figures 6A, 6B, 6C).

### Thrombocytes and Thrombocyte Functions

Thrombocytes with heterogeneous size, abnormal shape, and/or giant forms were observed on the peripheral blood smears of both the parents and patients. Giant and dysplastic thrombocytes were also evident in many patients’ bone marrow under light microscope (Figures 6A, 6B, 6C). 

**Supplemental Figure 1:**
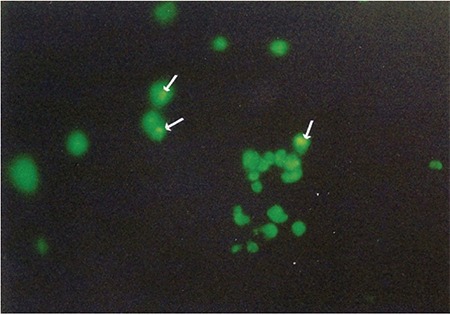
The platelet dense granules stained by mepacrine (arrow).

The mean dense granule number per thrombocyte was 2.01±1.19 (0.37-3.55) in the patients (n=12), 2.27±1.33 (0.22-4.47) in the parents (n=20), and 3.32±0.40 (2.78-3.82) in the healthy controls (n=5), and these were comparable with each other (p=0.147). However, the percentage of patients, parents, and controls who had fewer than 2 dense granules per thrombocyte was 50%, 35%, and 0% respectively (Supplemental Figure 1).

**Figure 7:**
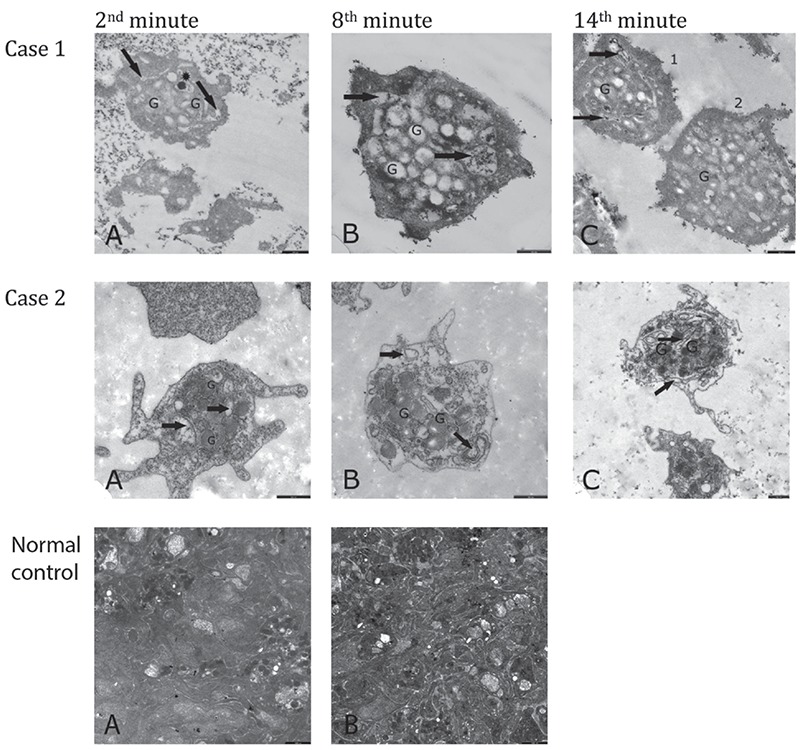
Electron microscopic images of the aggregating thrombocytes after addition of 2 µM ADP. All bars are 500 nm. Case 1 (patient BA, with unidentified mutation): A) 2^nd^ minute of aggregation: Thrombocytes are seen to have become close to each other; the granules (G) have centralized; open canalicular system is enlarged (↑); one dense granule is visible in enlarged open canalicular system (*). B) 8^th^ minute of aggregation: Platelets are still distant from each other. There are no platelets that fit tightly to each other. However, the granules (G) have centralized and have discharged their ingredients. Open canalicular system is enlarged and consists of residual secretion (↑). There are a few granules that have not evacuated their ingredients yet. C) 14^th^ minute of aggregation: The platelets are seen to have become closer but they are still apart from each other. The open canalicular system is enlarged and consists of some secretion (↑). There are and there are not undischarged granules (G) in thrombocytes 1 and 2, respectively. Case 2 (Patient ZG, with unidentified mutation): A) 2^nd^ minute of aggregation: Thrombocytes are seen to be apart from each other; the granules (G) have fairly centralized and are intact. B) 8^th^ minute of aggregation: The granules (G) are larger than normal and increased in number. They have centralized but have not discharged their contents yet. Pseudopods have developed. The open canalicular system has not enlarged yet (↑). C) 14^th^ minute of aggregation: The thrombocytes have not adhered to each other yet. The dense granules are distributed throughout the cytoplasm but the majority have not discharged their contents yet. Upper thrombocyte: The granules (G) are very large and increased in number. None of them have evacuated their contents. Open canalicular system (↑) is apparent. Normal Control: A) 2^nd^ minute of aggregation: Thrombocytes are seen to fit tightly together and display abundant pseudopods. They have released almost all of their granules. B) 8^th^ minute of aggregation: Thrombocytes fit tightly together. They have degranulated completely, except a few.

Ultrastructural examination showed that the thrombocytes had a reduced number of dense granules that were heterogeneous in size, shape, and composition. The open canalicular system (OCS) was enlarged and contained unevacuated components in patients (Figures 4H, 4I, 4J, 4K, and 7A - Case 2) and parents (Figures 5G and 5I).

In vitro bleeding time was prolonged in patients and parents by 37.5% and 18.8% with collagen-epinephrine cartridges and by 33.3% and 12.5% with collagen-ADP cartridges, respectively, vs. 0% in the control group with both cartridges. While in vitro bleeding times in patients and parents were comparable (p=0.293 and 0.233, respectively), only the in vitro bleeding time with collagen-ADP in patients was longer than in the control (p=0.031) (Supplemental Table 4).

Up to 63.6% and 44.4% of the aggregation results performed with various reactive substances in patients and their family members displayed abnormalities (Table 3; Supplementals Table 5 and 6).

Thrombocyte aggregation at the 2^nd^, 8^th^, and 14^th^ minutes under TEM (Figure 7) revealed a lack of adhesion and a lack of or inadequate secretion as also seen in Figures 4H, 4I, 4J, 4K, 5G, and 5I, with delayed or defective centralization, development of pseudopods, and/or secretion, abnormal degranulation, dissociation phenomenon [23,24], and abnormal amoeboid cytoplasmic protrusions (Supplemental Results).

There was inconsistency between the presence of hemorrhagic diathesis and abnormality of laboratory tests (aggregation tests, dense granule number in thrombocytes, in vitro bleeding time, thrombocyte ultrastructure) and vice versa. Not all these abnormalities coexisted all together (Table 3; Supplemental Table 6).

### Genetic Mutations

Fourteen of 15 patients and 9 of 21 parents were evaluated for genetic mutations. Patients had homozygous [c.130_131insA (p.W44*)] mutation in the second exon of the *HAX1* gene (n=6), heterozygous *ELANE* mutations [c.597+5G>A and c.416C>T (p.P139L) (n=2)], and homozygous *G6PC3* mutation [c.194A>C (p.E65A), n=2], which is a novel mutation in the literature and is predicted to be disease-causing by SIFT and MutationTaster in silico analysis software [in submission]. Five had unidentified mutations. No tested patient had *CSF3R* mutation. *ELANE *c.597+5G>A splicing mutation was predicted to be disease-causing by NNSPLICE, GeneSplicer, and Human Splicing Finder in silico prediction tools. 

Patients with *HAX1* displayed coexistent homozygous c.159T>C polymorphism in the second exon of the *HAX1 *gene. Three patients with other mutations were heterozygous for this polymorphism (Tables 1 and 3). Both the mother and father of AY, MNY, and HY and the mother of EÇ were found heterozygous for* HAX1* [c.130_131insA (p.W44*)]. The parents of the two patients with heterozygous *ELANE *mutation revealed no mutation in the *ELANE* gene and their buccal mucosa cells did not reveal mosaicism.

## Discussion

In this study, we showed that non-granulocytic blood cells were also affected and that morphologic and functional changes occurred in patients with SCN and in their non-neutropenic family members, and cell death mechanisms other than apoptosis also operated.

### Apoptosis and Secondary Necrosis in Granulocytic and Non-Granulocytic Cells

It has been reported that in SCN and other neutropenic states, accelerated apoptosis of bone marrow granulocytic progenitor cells [1,2,6,25,26,27,28,29,30,31] and lymphocyte apoptosis [7,8] took place through different mechanisms. In our study, apoptosis was demonstrated not only in granulocytes and lymphocytes but also in monocytes by elevated annexin V, ultrastructural appearance, and a pre-G1 peak in cell cycle analysis. The absence of a pre-G1 peak is not enough to exclude apoptosis [32]. Inconsistent elevations in CD95 and CD95 ligand pointed at a non-CD95-mediated apoptosis.

Only in the case of excessive apoptosis, during which the capacity of phagocytes to engulf apoptotic cells is reduced, do the uncleared apoptotic cells and fragments undergo secondary necrosis (delayed apoptotic clearance), which can provoke inflammation [33]. Our flow cytometric and microscopic findings revealed that apoptotic and necrotic cells coexisted in three cell lines in patients irrespective of the type of SCN mutation, and in their parents. 

We think that in our patients apoptotic and necrotic cells in the myeloid lineage activated macrophages and other phagocytes extensively (Figures 6A, 6B, 6C), inducing secretion of TNF-alpha, IL-1, IL-6, and IL-12 by activated macrophages, the latter exacerbating macrophage activation through stimulating IFN-gamma production [34,35]. High levels of TNF-alpha [36,37] in SCN patients and their non-neutropenic parents [36] and increased capacity of stimulated monocytes to produce TNF-alpha on stimulation through certain toll-like receptors [38,39] were reported before.

We consider the apoptosis in the non-granulocytic cells to be due to the high TNF-alpha levels, which can give rise to apoptosis in neutrophils [40,41], lymphocytes [42,43], monocytes [44], and thrombocytes [45] in various conditions [46] through TNF alpha-TNFR1 interaction (Supplemental Discussion, Text 1).

### Cytopenia in Non-Granulocytic Cells and RCS

Absence of lymphopenia (except 1 case), monocytopenia, and thrombocytopenia (Table 1; Supplemental Table 2) is apparently due to good compensation of the bone marrow of both the patients and the parents. 

However, it was striking that both patients and their parents had quantitative abnormalities in T lymphocytes and NK cells, regardless of the type of the SCN mutation carried by the patients. 

Abnormalities in B, cytotoxic T, NK, NKT, Th2, and Th7 cells were reported in SCN with *GFI-1* mutation, albinism-neutropenia syndromes, and Wiskott-Aldrich syndrome (WAS) [2,47,48,49,50,51]. None of our cases were clinically compatible with WAS or albinism-neutropenia syndromes. However, 2 SCN patients with *WAS *mutation were reported to have a reduced number of NK and CD4+ cells [7]. Interestingly, SCN patients with *ELANE *and unidentified mutations were reported to have normal numbers of NK cells that were less mature than those of normal controls [52]. NK cell deficiency and dysfunction was reported in some chronic neutropenia patients with morphological abnormalities [53]. In our cases, patients with NK levels lower than the normal range for age had *HAX1* (n=2) and *G6PC3* (n=2) mutations while patients with low CD3 (n=2) and CD4 (n=1) levels had unidentified mutations (Table 3). Mature neutrophils are reportedly necessary for NK cell development [52]. Reduced mature neutrophils may account for low levels of NK cells in the patient group but the reasons why not all SCN patients had low NK cells and why the parents who had low NK levels were not neutropenic require further investigations.

That the patients with low CD3+ and CD4+ lymphocytes (AG, ZG) were those with β-gal positivity of leukocytes suggests that the continual presence of circulating pro-inflammatory factors secreted by activated macrophages kept the immune system in a state of chronic low-level activation, giving rise to immunosenescence through loss of telomeric DNA with each S phase and therefore a decline in the number of T lymphocytes and no change or decline in overall lymphocyte and NK cells [54] during which inflammatory mediators secreted by senescent cells themselves contributed to immunosenescence [55,56] (we could not evaluate B lymphocytes) (Supplemental Discussion, Text 2). 

The presence of apoptosis together with RCS was reported in SCN [8] and in cell lines that had been administered cytotoxic drugs [57]. These cases (ZG and parents) in which individuals had not consumed cytotoxic drugs and were exposed to radiation may carry an unknown DNA-disrupting factor.

Cellular senescence is the state of irreversible cell cycle arrest, predominantly in the G1 phase [57,58,59,60,61,62,63,64], being dependent on (replicative senescence) or independent of telomeres (RCS) [60,61]. The latter is due to inappropriately expressed pro-proliferative genes [63], oncogenic mutations [62], DNA-damaging drugs, or gamma irradiation [57,58]. 

The senescence-like phenotype is characterized by reorganization of heterochromatin [65,66], formation of fragmented nuclei, polyploidy, and enlarged and flattened cell shape, along with expression of SA-β-gal positivity [15,57] and alterations in the cell cycle [65].

### Dysplasia of Hematopoietic Cells

In our study, as reported previously [8,10], dysplasia was noted not only in the neutrophil series but also in the monocyte, megakaryocyte, lymphocyte, and eosinophil series in all patients and parents to various degrees. Some dysplasia parameters overlap with the senescence phenotype; however, restriction of RCS to a few cases in our study showed that RCS only partially affected the development of dysplasia. Our previous studies point at the role of inflammatory cytokines to cause dysplasia [36,37] in patients with autoimmune disorders, acute infections, and hemophagocytic histiocytosis [67,56]. The pro-inflammatory cytokines secreted by activated macrophages can destroy the bone marrow microenvironment and hematopoietic stem cell niches by activating innate immune cells [55] and give rise to hematopoietic stem cell dysfunction, dyshematopoiesis, and thus dysplastic hematopoietic cells.

Morphologic abnormalities due to abnormal differentiation in myeloid cells are also encountered in congenital, cyclic, dysgranulopoietic neutropenia cases [7,10,66,68] with or without the* WAS, GFI-1,* and* G6PC3* mutations [1,7], in myelodysplastic syndrome, and in a number of non-malignant disorders [16,67].

### Dysmegakaryopoiesis and Hemorrhagic Diathesis

That the presence of nearly no normal megakaryocytes in our patients and that nearly all megakaryocytes displayed characteristics of naked megakaryocyte nuclei, emperipolesis, or abnormal morphology like peripheral vacuolization (showing non-classical apoptosis: para-apoptosis) and directly destructed megakaryocytes (showing necrosis) and presence of many stage 1 megakaryocytes (megakaryoblasts), some of which were aberrantly releasing thrombocytes, imply defective megakaryocyte maturation, heavy intramedullary premature cell death of megakaryocytes, and increased megakaryopoiesis [14,69,70,71,72,73] (Supplemental Discussion, Text 3).

Defective maturation in megakaryocytes is also expected to be due to the increased levels of pro-inflammatory cytokines, which can destroy the bone marrow microenvironment and hematopoietic stem cell niches [55]. Therefore, thrombocytes derived from megakaryocytes with defective maturation are also expected to be functionally abnormal. 

Hence, in our cases, we noted a combination of thrombocyte functional defects and in a few of them a low number of mepacrine-labeled dense granules reminiscent of a delta storage pool defect. The ultrastructural view of aggregating thrombocytes (Figure 7; Supplemental Discussion, Text 3) may reflect defective transmission. From all these aspects, the findings of our patients resemble the thrombocyte disorders encountered in leukemia, refractory anemia, cystinosis, and others [19,23,24,74,75]. 

Hemorrhagic diathesis is a common finding of albinism-neutropenia syndromes like Chediak-Higashi syndrome, Hermansky-Pudlak syndrome type 2, Griselli’s syndrome type 2, Cohen’s syndrome, and p14 deficiency [51] but has not been reported in SCN [76] before. 

Our results, at the same time, confirmed that not all patients with thrombocyte aggregation defects display laboratory evidence [18,77,78] and the most reliable tool to show thrombocyte aggregation defect is electron microscopic evaluation [78,79,80].

### Parents

No parent had cytopenia; however, apoptosis and secondary necrosis to various degrees in granulocytes, monocytes, and lymphocytes with the presence of dysplasia, decreased NK cells, and abnormalities in thrombocyte functions in most of the parents and RCS in one suggest that the parents were also affected by the same genetic abnormality but the cell loss was well compensated by the proliferating compartment. However, only the parents of patients with *ELANE* and those of most patients with *HAX1* could be evaluated genetically. 

For the parents of the patients with homozygous *HAX1 *mutation (AY, MNY, HY, EÇ) who were heterozygous for the same mutation, we think that cell loss took place through one mutant allele, just like in their children. The apoptotic hematopoietic cells (lymphocytes, neutrophils, monocytes) were the mutant cells that were lost early [81,82], but normal hematopoiesis compensated for the cell loss when the other allele was normal. 

As for the parents of patients with *ELANE *mutation (RT, NBÖ), the absence of* ELANE* mutation in the parents’ peripheral blood cells led us to consider that the parents were either mosaic for the mutation or actually normal and their children were sporadic cases of *ELANE* mutation. Hence, a number of phenotypically healthy parents were shown to harbor somatic [83], only germline [3], or both somatic and germline [84] mosaicism of *ELANE* mutation. On the other hand, most of the sporadic cases of SCN were reported to have* ELANE* mutations [85].

That we could not detect mosaicism in the buccal mucosa cells of the parents does not rule out mosaicism definitively. A search for mutant alleles in various other cell types like skin or sperm of the father, preferably using more sensitive mutation analysis methods, might have proved mosaicism, like in the reported cases [3,86] in which the mutant allele was negative in DNA from neutrophils, buccal mucosa, and/or lymphocytes and was detected only in spermatozoa. However, the parents felt uneasy about being tested any further. 

Additionally, that the parents of patient RT had normal blood cell counts but high cell death parameters in lymphocytes, granulocytes, and monocytes like the parents with heterozygous *HAX1* suggested that they were very probably affected by the same mutation in the same gene,* ELANE*, as their children. However, we cannot exclude the possibility that patient RT was a sporadic case of *ELANE *mutation and did not additionally harbor any other untested/unidentified neutropenia mutation [87] and that his parents were carriers of this mutation. On the other hand, similar death parameters in the hematopoietic cells of the parents of NBÖ to those of the controls suggested that the *ELANE *mutation in NBÖ could be sporadic. As a second possibility, both of the parents of NBÖ might have cyclic hematopoiesis with consecutive normal and abnormal hematopoiesis [88], just like in the mother of a patient with *JAGN1* mutation who we followed before [8,10, unpublished data]. However, we could not exclude an unidentified mosaicism for the parents of NBÖ definitively due to the same reasons. 

As for the parents of patients with *G6PC3* (OSK, MeK) and unidentified mutations (AO, BA, AG, ZG, KŞ), only two siblings with unidentified mutation (AG, ZG) and their parents could be evaluated for cell death parameters and both the parents’ blood cells (lymphocytes, granulocytes, monocytes) showed apoptosis and necrosis similar to that of their children. Therefore, we think that the parents of patients with other recessive SCN gene mutations (like *G6PC3* and at least some of the unidentified mutations) might be heterozygous for the same genetic defect, like in the parents who were heterozygous for *HAX1.*

We think that the modifying effects of other genes or factors [3,89] and any other accompanying neutropenia mutations [87] and many other factors that play roles in the transmission of disease, including the 159T>C polymorphism in the same exon of *HAX1* mutation in patients with *HAX1* and other mutations as in other cases [90,91], both in the patients and parents, need to be evaluated in further studies.

The gingival enlargement and oral aphthae of the parents in the present study were thought to be possibly due to dysfunctional neutrophils, which were dysplastic at the same time, like a non-neutropenic mother of a patient with *JAGN1* mutation who we followed before and had apoptosis in addition to morphological and functional abnormalities in neutrophils, lymphocytes, and thrombocytes with low levels of myeloperoxidase and defective chemotaxis [8,10, unpublished data].

Easy bruising and gingival bleeding of the parents were attributed to defective thrombocyte functions stemming from defective megakaryopoiesis. 

We attribute apoptosis and secondary necrosis in the myeloid lineage of the non-neutropenic parents to the aforementioned genetic abnormalities relevant to SCN, while those in the non-granulocytic cells to the high TNF-alpha levels [36] that can give rise to apoptosis in blood cells [40,41,42,43,44,45,46], as discussed for children with SCN.

## Conclusion

Apoptosis and secondary necrosis in non-granulocytic cell lines, dysplasia of blood cells with/without RCS, and disturbances in lymphocyte subsets and thrombocyte functions were observed in patients with congenital neutropenia and their non-neutropenic parents. Additionally, bone marrow of the patients showed increased phagocytic activity and striking dysmegakaryopoiesis. (Table 4). This study shows that abnormalities in lymphocyte subsets and hemorrhagic diathesis are not restricted to albinism-neutropenia syndromes, as current wisdom holds, but are also encountered in SCN. 

Moreover, our findings suggest that the pluripotent hematopoietic stem cells in SCN are defective irrespective of the genetic etiology, in contrast to the current thinking that understands the main defect as residing in the progenitor myeloid cells [1,2,6], and myeloid transcriptional factors [92,93] 

### Study Limitations

The main limitation of our study was that not all sub-studies could be performed for all cases due to daily limitations of our laboratory facilities. Including idiopathic neutropenic patients as a separate control group could have helped evaluate the results more extensively, although these patients were beyond the scope of this study. In spite of this, we believe that our results may lead to further in vivo and in vitro studies involving pluripotent hematopoietic stem cells in SCN so as to better understand the underlying physiopathology. Additionally, the presence of the same abnormalities in non-neutropenic parents shows that the phenotype-genotype relationship is another field that requires further evaluation.

## Figures and Tables

**Table 1:**
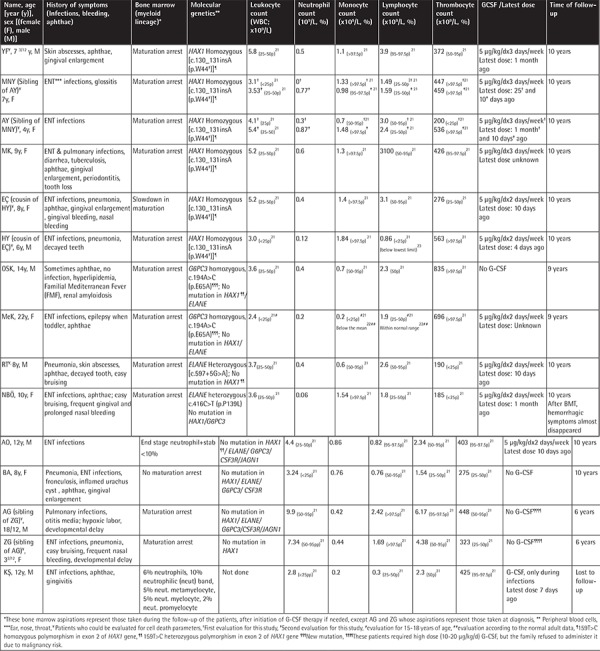
General characteristics of the patients.

**Table 2:**
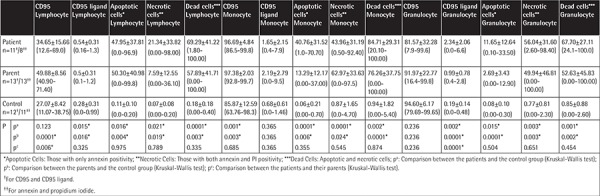
CD95, CD95 ligand, and annexin levels on the lymphocytes, granulocytes, and monocytes of the patients and their parents (%).

**Table 3:**
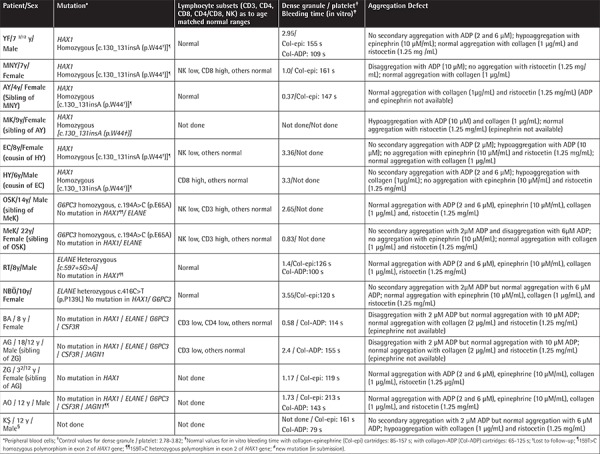
Laboratory parameters of patients.

**Table 4:**
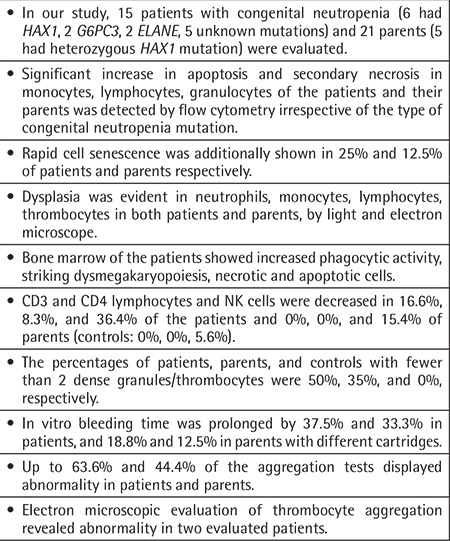
Summary of the findings.

**Supplemental Table 1:**
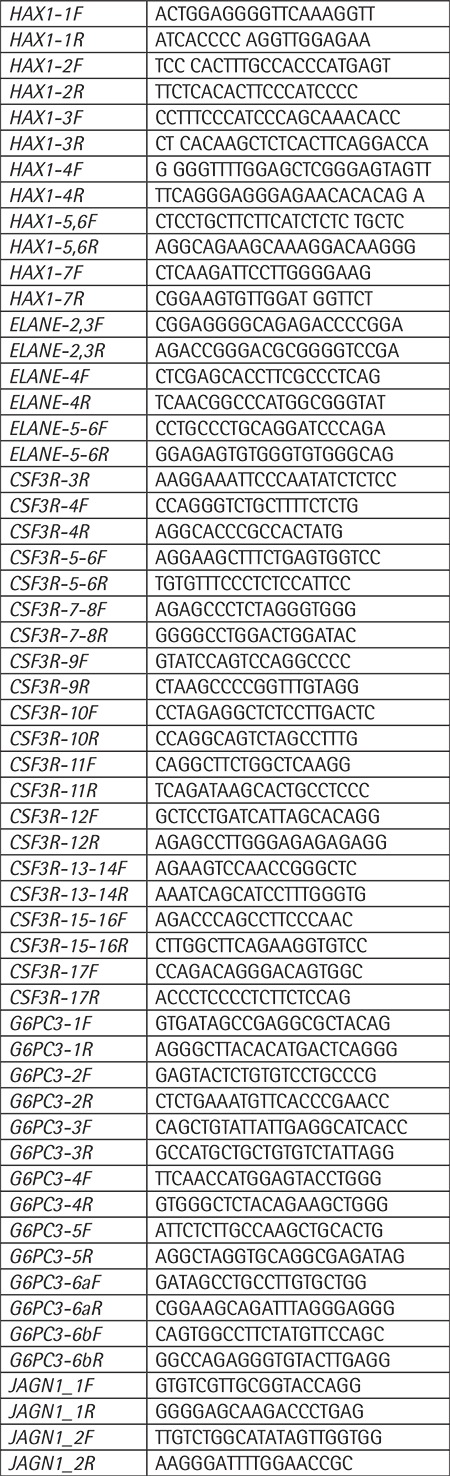
Primers used for the sequencing of the coding regions of *HAX1, ELANE, G6PC3, CSF3R, JAGN1* genes.

**Supplemental Table 2:**
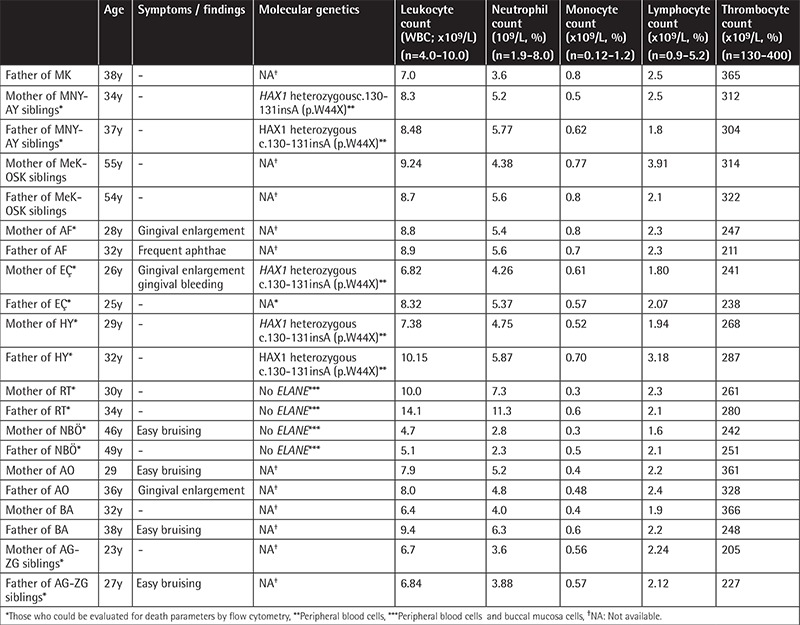
Characteristics of the parents.

**Supplemental Table 3:**
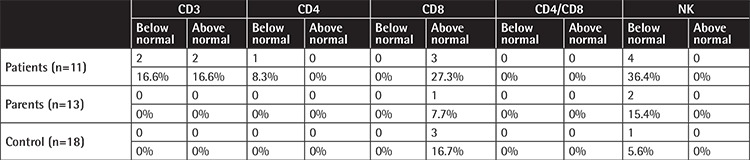
The number and percentage of the patients and their parents whose lymphocyte subset levels were below and above age matched normal range.

**Supplemental Table 4:**
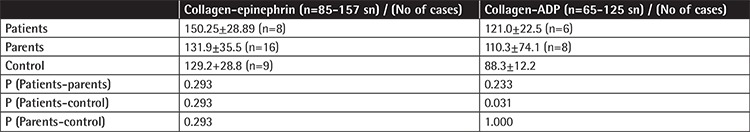
In vitro bleeding time of the cases.

**Supplemental Table 5:**
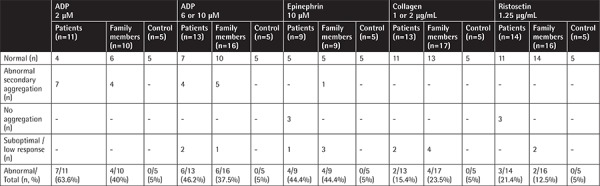
Platelet aggregation responses to various agonists.

**Supplemental Table 6:**
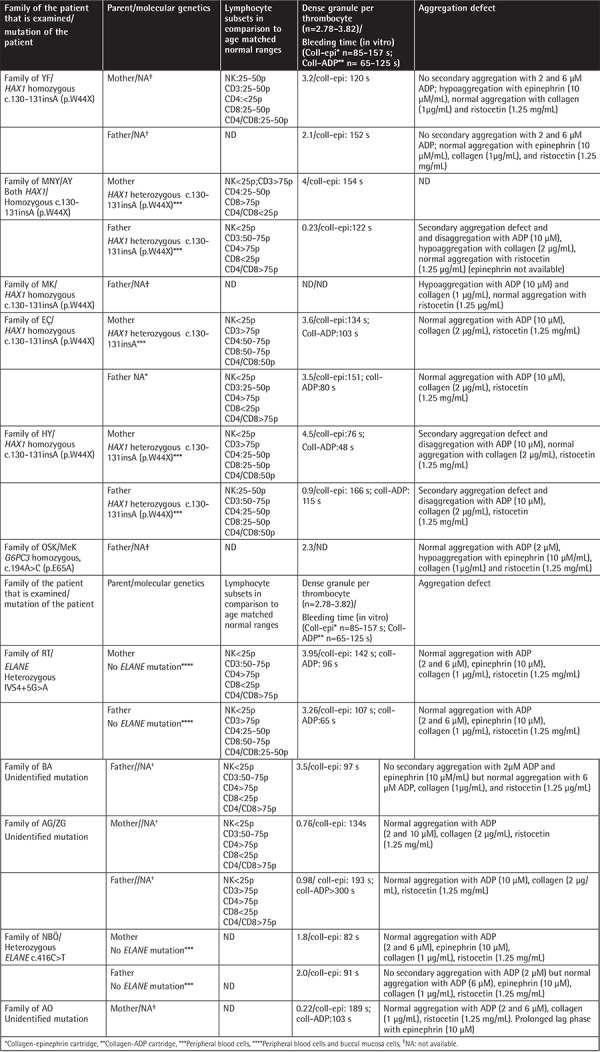
Laboratory parameters of the parents.

**Figure 2.A1:**
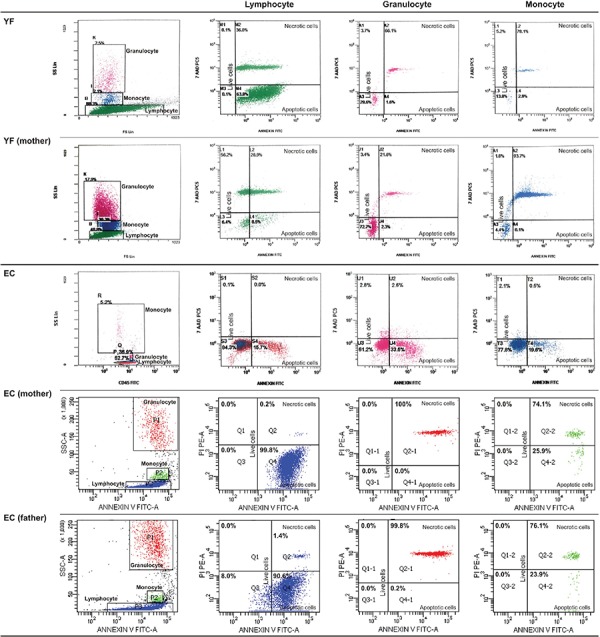
FACS gating and flow cytometric graphics of annexin and PI/7-AAD of lymphocytes, monocytes, and granulocytes of patients with congenital neutropenia and their parents. 2A1 and 2A2: Those of patients with *HAX1* mutation (YF, EC, AY, MNY, HY) and their parents. The mother and father of AY, MY, and HY and the mother of EÇ were heterozygous for *HAX1*.

**Figure 2.A2:**
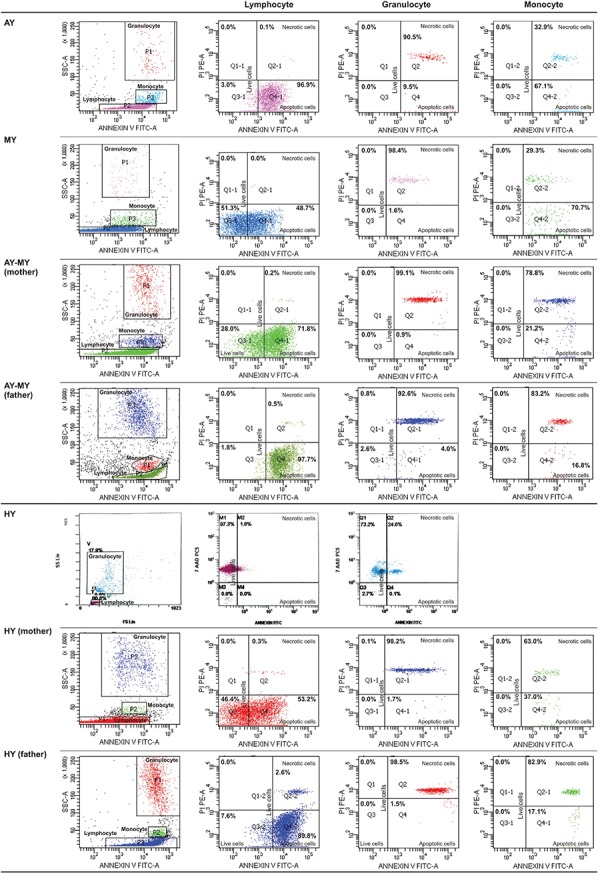
FACS gating and flow cytometric graphics of annexin and PI/7-AAD of lymphocytes, monocytes, and granulocytes of patients with congenital neutropenia and their parents. 2A1 and 2A2: Those of patients with *HAX1* mutation (YF, EC, AY, MNY, HY) and their parents. The mother and father of AY, MY, and HY and the mother of EÇ were heterozygous for *HAX1*.

**Figure 2.B:**
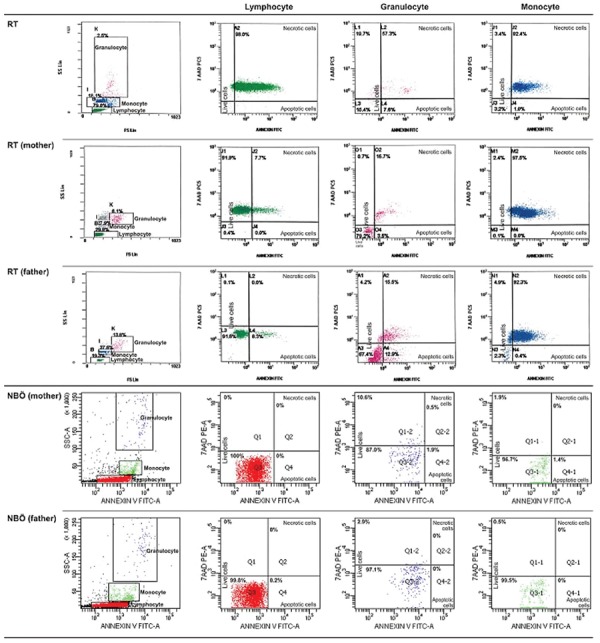
FACS gating and flow cytometric graphics of annexin and PI/7-AAD of lymphocytes, monocytes, and granulocytes of patients with congenital neutropenia and their parents. 2B: Those of RT with *ELANE* mutation and his parents and parents of NBÖ with *ELANE* mutation. The cells of patient NBÖ could not be evaluated. Neither of the parents had *ELANE* mutation in peripheral lymphocytes or buccal mucosa.

**Figure 2.C:**
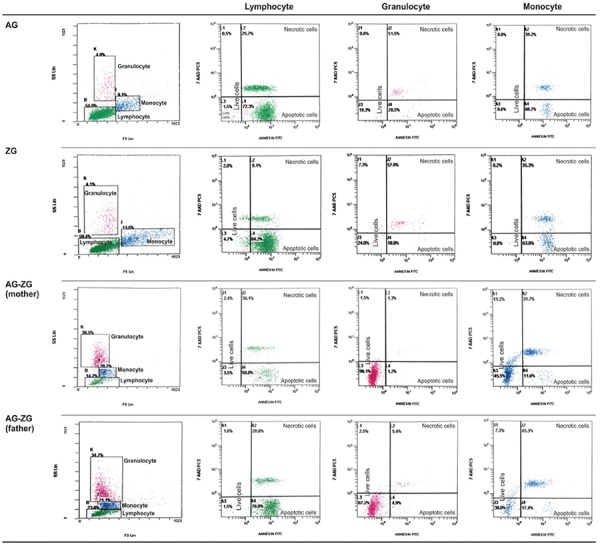
FACS gating and flow cytometric graphics of annexin and PI/7-AAD of lymphocytes, monocytes, and granulocytes of patients with congenital neutropenia and their parents. 2C: Those of two siblings with congenital neutropenia with unidentified mutation and their parents (AG, ZG). 2D: Those of one of the healthy volunteers.

**Figure 2.D:**
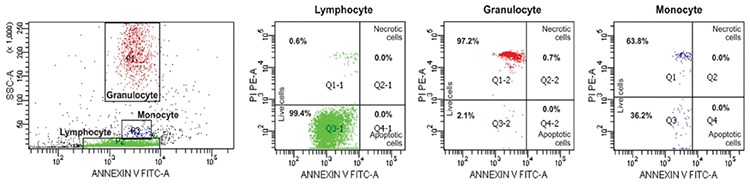
FACS gating and flow cytometric graphics of annexin and PI/7-AAD of lymphocytes, monocytes, and granulocytes of patients with congenital neutropenia and their parents. 2C: Those of two siblings with congenital neutropenia with unidentified mutation and their parents (AG, ZG). 2D: Those of one of the healthy volunteers.

**Figure 6.A:**
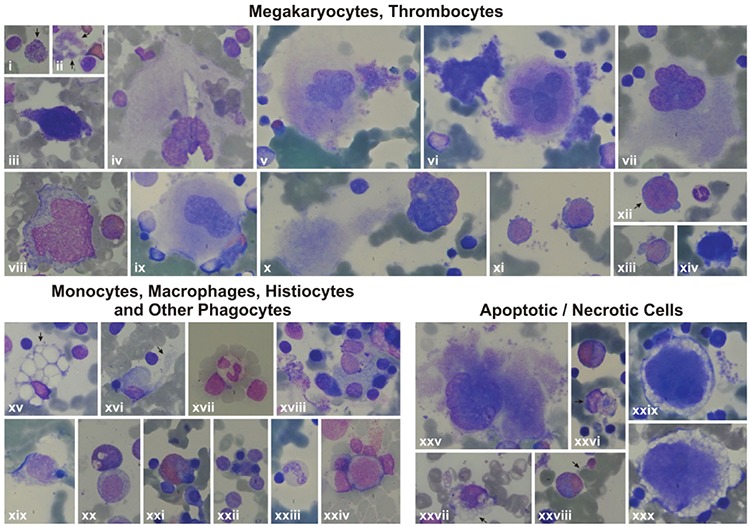
Features of some bone marrow cells (megakaryocytes, thrombocytes, monocytes, macrophages, histiocytes, other phagocytes, and apoptotic/necrotic cells) of the patients by light microscope. 6A: Features of some bone marrow cells from the patients with *HAX1* mutation. i, xxvii, xxviii): Patient AY; ii) Patient EÇ; iii, iv, viii, xvi, xxx) Patient MNY; v, vi, x, xii, xv, xvii, xviii, xxi, xxii, xxiii, xxiv, xxvi) Patient MK; vii, xiv, xxix) Patient YF; xi, xii, xiii, xix, xx, xxv) Patient HY (1000^x^). i) Giant thrombocyte; ii) dysplastic thrombocytes; iii, xi, xii, xiii, xiv) Megakaryoblasts with nucleo-cytoplasmic asynchrony; iv, v, vi, ix) senescent megakaryocytes undergoing the process of being naked megakaryocyte nucleus and naked megakaryocyte cytoplasm, just as producing (v, vi) or after completing production of thrombocytes (iv, vii, ix), with emperipolesis of other bone marrow cells (iv, ix); viii) Abnormal megakaryocyte; x) a megakaryocyte that has just developed into a naked megakaryocyte nucleus and naked megakaryocyte cytoplasm. xv) A macrophage full of fat (Gaucher-like cell); xvi, xix, xxii, xxiv) monocytes that are phagocytosing various mononuclear cells; xx) a monocyte phagocytosing an apoptotic cell; xvii, xxi, xxiii) other phagocytes like a neutrophil (xvii), eosinophilic myelocyte (xxi), and stab (xxiii) that are phagocytosing other bone marrow cells; xxv) A megakaryocyte undergoing necrosis; xxix, xxx) megakaryocytes undergoing apoptosis; xxvi) a monocyte that had performed phagocytosis and is undergoing necrosis; xxvii) a monocyte undergoing necrosis; xxviii) an eosinophilic myelocyte with an apoptotic body attached to the cell.

**Figure 6.B:**
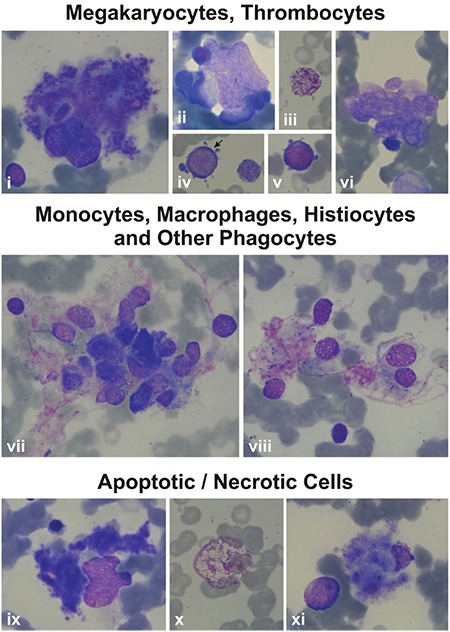
Features of some bone marrow cells (megakaryocytes, thrombocytes, monocytes, macrophages, histiocytes, other phagocytes, and apoptotic/necrotic cells) of the patients by light microscope. Features of some bone marrow cells from the patients with *ELANE* mutation. i, iii, iv, vii, viii, ix, x, xi) Patient NBÖ (1000^x^); ii, iv, vi) Patient RT (1000^x^). i) A degenerating dysplastic megakaryocyte; ii) a mononuclear megakaryocyte undergoing emperipolesis; iii) a giant thrombocyte; iv, v) megakaryoblasts with nucleocytoplasmic asynchrony; vi) naked megakaryocyte nuclei that could not transform to unique nuclei. vii, viii) Histiocytes that phagocytosed many bone marrow cells; ix) A megakaryocyte that is just about to undergo necrosis; x) a ghost-like cell degenerating through secondary necrosis; xi) a necrotic megakaryocyte.

**Figure 6.C:**
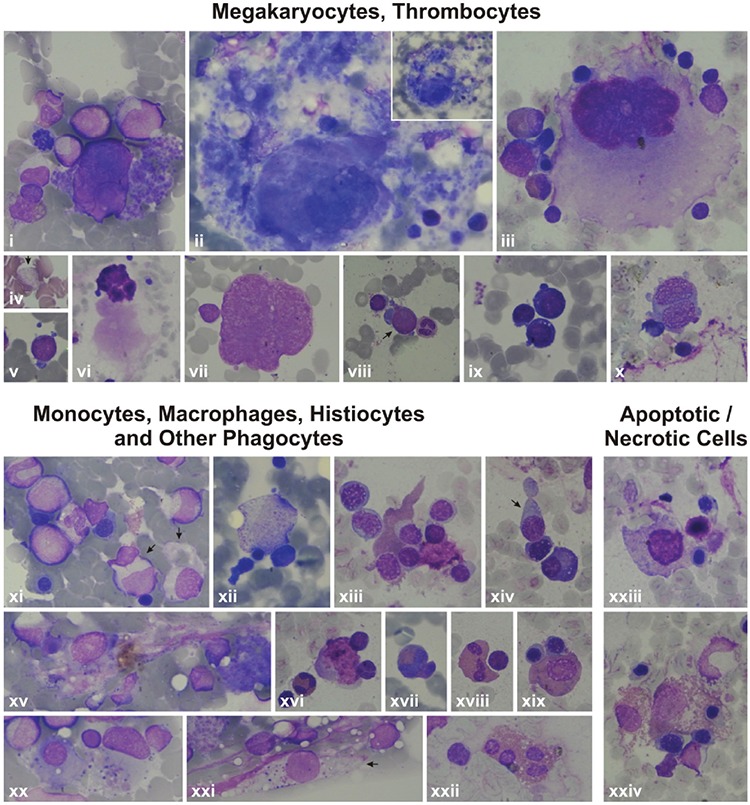
Features of some bone marrow cells (megakaryocytes, thrombocytes, monocytes, macrophages, histiocytes, other phagocytes, and apoptotic/necrotic cells) of the patients by light microscope. 6C: Features of some bone marrow cells from the patients with unidentified mutations. i, v, vii, viii, xi, xv, xx, xxi) Patient AO; iii, vi, ix, x, xiii, xiv, xvi, xviii, xix, xxii, xxiii, xxiv) Patient ZG; iv) Patient BA; ii, xii, xvii) Patient KŞ (1000^x^, except Figure vi, which is presented at 400^x^). i) A megakaryocyte with nucleo-cytoplasmic asynchrony; ii, iii) senescent megakaryocytes undergoing the process of transformation to naked megakaryocyte nucleus and naked megakaryocyte cytoplasm, just as producing thrombocytes (ii) or after finishing production of thrombocytes (iii) or with emperipolesis (iii); iv) a giant thrombocyte; v, viii, ix) megakaryoblasts; vii) a megakaryocyte that is just transforming to naked megakaryocyte nucleus and naked megakaryocyte cytoplasm; vii) a naked megakaryocyte nucleus; x) a dysplastic megakaryocyte with two nuclei and scanty cytoplasm but thrombocyte production; xi, xii, xvi, xix) Monocytes (xi, xiv), macrophages (xii, xvi, xix) that had phagocytosed or are phagocytosing bone marrow cells with eosinophilic cytoplasm (xix) or pseudopods (xiv); xv, xx, xxi) histiocytes that have been phagocytizing bone marrow cells (xv, xx) and/or consist of basophilic debris, which gives the appearance of sea blue-like histiocytes (xv, xx, xxi); xvii, xviii) phagocytosis of other phagocytes like eosinophils (xviii) or eosinophil metamyelocytes (xvii); xxiii, xxiv) Macrophages with normal (xxiii) or eosinophilic (xxiv) cytoplasm that had performed hemophagocytosis and are undergoing necrosis now.
