# Supplementary material for: Both Granulocytic and Non-Granulocytic Blood Cells Are Affected in Patients with Severe Congenital Neutropenia and Their Non-Neutropenic Family Members: An Evaluation of Morphology, Function, and Cell Death
Source: Turk J Haematol. 2018 Nov 13;35(4):229–59. doi: 10.4274/tjh.2017.0160 (PMC6256814; doi:10.4274/tjh.2017.0160)
Supplement: Related texts

# Addendum: Appendix: Supplemental Materials and Methods

## Mutation Analysis

Mutation analyses of the *HAX1, ELANE, CSF3R*, and *G6PC3* genes were performed by standard techniques using a DNA sequencing kit (PerkinElmer, Foster City, CA, USA) and the ABI Prism 3100 sequence analyzer (Applied Biosystems, Foster City, CA, USA).

## Reference accession number of the genes

*HAX1* (NM_006118)

*ELANE* (NM_001972)

*CSF3R* (NM_156039)

*G6PC3* (NM_138387)

*JAGN1* (NM_032492)

## Evaluation of Cellular Morphology

The peripheric blood cells were evaluated by light (Nikon Eclipse E400, Nikon Corporation, Japan) and transmission electron microscope (TEM) (LEO 906E, Zeiss, Germany) for apoptosis and dysmorphism [1,2,3,4], in a blinded fashion. One hundred neutrophils of the parents (except YF’s mother whose peripheral blood smear enabled us to evaluate only 50 neutrophils) and control group were scored for dysmorphism under light microscope (1000^x^). Each dysmorphic feature shown by an individual neutrophil was given one score and the total score for a definite dysmorphic feature was presented as ‘%’. Only for YF’s mother the total score for a definite dysmorphic feature was multiplied by two. The patients’ neutrophils could not be scored due to neutropenia. The photographs of the representative cells with dysmorphic features under electron microscope were taken and presented.

## Evaluation of the Thrombocytes of the Patients and the Parents

*In vitro bleeding time: *This was measured by a PFA−100 instrument (Dade Behring Marburg GmbH, Marburg, Germany) using collagen-epinephrine and/or collagen-ADP cartridges [5].

*Turbidimetric aggregation tests:* A Chrono-Log 560 Ca aggregometer was used with minor modifications (Chrono-Log Corporation, Havertown, PA, USA) [5]. Thrombocyte aggregation was studied with collagen (1 and 2 µg/mL), ristocetin (1.25 mg/mL), ADP (2, 6, 10 µM), and epinephrine (10 µM).

*Mepacrine labeling of dense granules:* Thrombocyte-rich plasma was prepared and stained with mepacrine (1 µM; Sigma, St. Louis, MO, USA) as described previously [6,7]. The greenish-yellow fluorescent dense granules were visualized under a fluorescent microscope (Zeiss Axoscope; Zeiss, Thornwood, NY, USA). The dense granules were counted in 84-106 platelets for each sample. The results were presented as the mean dense granule number per thrombocyte [6,7].

*Electron microscopic evaluation of aggregation: *After adding 2 µM ADP to thrombocyte-rich plasma obtained from two patients (BA, ZG), samples were removed at the 2^nd^, 8^th^, and 14^th^minutes and were prepared for TEM [6].

## Statistics

We used SPSS 15.0 to evaluate the data we obtained (SPSS Inc., Chicago, IL, USA). A normality test was performed to determine if the data were distributed in a normal fashion. To compare evenly and unevenly distributed data, we used the independent samples t-test and the Mann-Whitney U test (for two groups) and One-way ANOVA and the Kruskal-Wallis H test with Bonferroni correction (for more than two groups). We used the Scheffe test in the one-way ANOVA testing to determine which groups had differences between themselves. One-sample t-tests were used to evaluate whether the means of the variables differed from the means of the reference group. Values of p<0.05 and p<0.017 were considered significant for inter-group comparison and the Kruskal-Wallis H test, respectively.

## Supplemental Results

## Ultrastructural View of Aggregating Thrombocytes

Aggregation tests under TEM revealed the following abnormalities: **1) Lack of adhesion: **At the 2^nd^, 8^th^, and 14^th^ minutes of the aggregation test, the thrombocytes were still separate from each other. At the second minute, the granules were fairly or completely centralized with no visible pseudopods, whereas normal thrombocytes fit together tightly, displayed abundant blunt pseudopods, and had released almost all of their granules (Case 1, Case 2, and control). **2) Lack of or inadequate secretion:** Organelle centralization took place but the organelles later became dispersed in the cytoplasm, without undergoing secretion and with unevacuated large granules and no enlargement in the OCS (Case 2) or having made partial secretion with enlarged OCS full of unsecreted material (Cases 1 and 2). **3) Delay in all stages of aggregation:** Each stage of aggregation (centralization, development of pseudopods, and/or absent or impartial secretion) took place with delay (Case 1, Case 2, and control). **4) Abnormal degranulation:** Unlike in normal degranulation, in which the dissolution of the granule’s membrane is followed by evacuation of granule contents into the OCS and canalicular dilation, in some samples of the patients the dense granule was evident in the OCS, without membrane dissolution (Case 1). **5) Dissociation phenomenon: **The patients’ platelets showed the dissociation phenomenon, a disruption of normal aggregation that normally takes place in a coordinated sequence of pseudopod formation, change in shape, clustering of central granules, degranulation, and canalicular dilation [8,9]. In our cases, all of these changes took place without the adherence of thrombocytes to each other. We observed OCS enlargement and secretion of some material taking place before the degranulation phase ended (Case 1). **6) Abnormal cytoplasmic protrusions:** The cytoplasmic protrusions in normal aggregating thrombocytes were thin, whereas in our patients they were amoeboid (Cases 1 and 2).

## Clinical Correlations Between Hemorrhagic Diathesis and Laboratory Parameters

It was striking that not all cases with a history of hemorrhagic diathesis displayed abnormal aggregation test results, low dense granule number in thrombocytes, or prolonged in vitro bleeding time, whereas many cases without any history of hemorrhagic diathesis displayed abnormal aggregation test results or low dense granule number in thrombocytes or prolonged bleeding time or abnormal ultrastructure of thrombocytes. Not all of these abnormalities coexisted all together (Table 2; Supplemental Table 5).

## Supplemental Discussion

## Text 1

In the steady state apoptotic cells are rarely encountered under physiological conditions and the removal of the apoptotic cells is fast and promotes an anti-inflammatory response [10]. However, in the case of substantial and excessive apoptosis during which the capacity of phagocytes to engulf apoptotic cells is reduced, the uncleared apoptotic cells and fragments undergo secondary necrosis (delayed apoptotic clearance) which can provoke inflammation or autoimmunity. Macrophages that ingest necrotic cells cause increased T cell proliferation [10].

Hence, the flow cytometric graphics show that apoptotic and necrotic cells coexisted in all three cell lines, confirming that necrosis was due to excessive apoptosis. Hence, phagocytosis of both necrotic and apoptotic cells were shown in the bone marrow of the patients.

However, presence of excessive apoptosis and secondary necrosis not only in the patients’ neutrophils but in all other cell lines (lymphocytic and monocytic) regardless of the mutation type of congenital neutropenia and their existence even in the non-neutropenic parents of the congenital neutropenia patients seem unreliable at first glance.

We think that macrophages of our patients were activated by increased apoptosis in the myeloid lineage, and the TNF-alpha secreted by activated macrophages gave rise to increased apoptosis (and secondary necrosis) in all cell lineages. Hence, striking phagocytic activity of various phagocytic cells (histiocytes, monocytes, macrophages, neutrophils, bands and even eosinophils and eosinophil myelocytes), mainly being monocytic cells, and our previous findings showing elevated TNF-alpha which is mainly secreted by activated macrophages in a group of congenital neutropenia patients, most of whom also enrolled in this study [11], confirm our explanation.

Additionally, studies of other colleagues showing enhanced stimulating effect of monocyte activation through toll-like receptors on TNF-alpha production [12,13], and increased baseline cytokines including TNF-alpha in children with congenital neutropenia [14] also draw attention to the role of TNF-alpha and other inflammatory cytokines in congenital neutropenia.

Monocytes are activated by microbial products as well as molecules expressed or released by dying or stressed cells through toll-like receptors, interferon gamma, Th1 cells or complement fragments [15]; additionally removal mechanisms of necrotic and apoptotic cells may overlap [10]. Once activated, macrophages secrete TNF-alpha, IL-1, IL-6 and IL-12, the latter of which is also secreted by dendritic cells and stimulates IFN-gamma production by NK and T cells thereby exacerbating macrophage activation [15,16]. Hence, elevations of pro-inflammatory cytokines in congenital neutropenia were reported before [11,14].

TNF-alpha which is mainly secreted by macrophages is a well known cytokine to give rise to apoptosis in neutrophils [17,18], lymphocytes [19,20], monocytes [21], and thrombocytes [22] in various conditions like sepsis, HLH [23], stroke, and ankylosing spondilitis and secreted mainly by macrophages and through TNF alpha and TNFR1 interaction.

Therefore, the factor that stimulates macrophage activation in our patients, none of whom had any major infection other than gingivitis or aphthous stomatitis at the time of evaluation, seems to be excessive apoptosis (thereby secondary necrosis) in the granulocytic lineage due to various mutations distrupting normal granulopoiesis. Apoptosis and secondary necrosis of other cell lines like lymphocytes and monocytes as shown by flow cytometry, and in megakaryocytes as shown by light microscopy are thought to be secondary to increased TNF-alpha through excessive macrophage activation.

## Text 2

On the other hand, RCS in 25% and 12.5% of the evaluated patients and parents, documented by b-gal positivity of leukocytes may be another reason for quantitative abnormalities in T lymphocytes. That the patients with low CD3+ and CD4+ lymphocytes (sibling patients AG, ZG) were those with b-gal positivity of leukocytes showing RCS and with normal NK levels suggests a relationship between T-lymphopenia and RCS. Presence of immune activation in our patients documented by increased phagocytic activity in their bone marrow suggests that continual presence of circulating pro-inflammatory aforementioned factors secreted by activated macrophages had kept the immune system in a state of chronic low-level activation giving rise to an immunosenescence through loss of telomeric DNA with each S phase, and therefore a decline in the absolute number of T and B lymphocytes, with no change in overall lymphocyte count but a relative increase in NK cells [24] (in our patients we could not evaluate B lymphocytes). A set of mediators including inflammatory mediators secreted by senescent cells themselves contribute to the chronic inflammation [25] and the loss of telomeres. Likewise, a previous study of ours showed RCS (b-gal positivity) of leukocytes in active autoimmune disorders [systemic lupus erythematasus (SLE), juvenile rhematoid arthritis (JRA), and immune thrombocytopenic purpura (ITP)], in all of which pro-inflammatory cytokines are high [26].

## Text 3

## Dysmegakaryopoiesis and Hemorrhagic Diathesis

In our patients one of the most prominent findings was abnormal megakaryopoiesis evidenced by naked megakaryocyte nuclei (NMN), megakaryocyte emperipolesis, megakaryocytes with features of apoptosis, paraapoptosis, necrosis, and abnormalities in thrombocyte aggregation.

Naked megakaryocyte nuclei form after progressive folding of lobulated nucleus itself and then contracting (pyknosis), in order to reach the lowest volume that the megakaryocyte can occupy. The surrounding small rim of cytoplasm contains no characteristic MK granules or demarcation membranes. In physiological conditions, NMN are senescent cells which completed thrombocyte shedding and are few in normal bone marrow. However, large numbers of NMN in the bone marrow, like in myeloproliferative diseases and AIDS show defective maturation, a heavy intramedullary premature cell death of megakaryocytes and increased megakaryopoies [27, 28].

Emperipolesis [29,30] is a random passage of one cell through the cytoplasm of another one with no physiological change in neither of them. It is most frequent in megakaryocytes and the most engulfed cell types are neutrophils and erythrocytes, as in our patients.

Naked megakaryocyte nuclei [27] and megakaryocytes with emperipolesis [29] were shown to display the features of paraapoptosis (non classical apoptosis).

While apoptosis is characterized by margination of condensed chromatin, nuclear fragmentation and formation of apoptotic bodies, paraapoptosis of megakaryocytes is characterized by cytoplasmic vacuolization due to mitochondrial swelling and distended demarcation membrane system (DMS), condensed nuclear chromatin with no early chromatin margination and no surface blebbing and apoptotic body formation. All paraapoptotic megakaryocytes were shown to have an intact, thickened peripheral zone which seemed to contain no functional cellular material, like organelles or DMS and their cytoplasm did not contain thrombocyte territories [31], like in our patients.

Additionally presence of many stage 1 megakaryocytes (megakaryoblasts), some of which are aberrantly releasing thrombocytes, unlike what is expected, also show dysmegakaryopoiesis [32].

That presence of nearly no normal megakaryocytes in our patients and that nearly all megakaryocytes displayed characteristics of NMN, emperipolesis or abnormal morphology like peripheral vacuolization (showing non-classical apoptosis: paraapoptosis and directly destructed megakaryocytes (showing necrosis) imply defective megakaryocyte maturation, a heavy intramedullary premature cell death of megakaryocytes and increased megakaryopoiesis.

Defective maturation in megakaryocytes is also expected to be due to the increased levels of aforementioned proinflammatory cytokines secreted by and after macrophage activation which can destroy the bone marrow microenvironment and hematopoietic stem cell niches [25] giving rise to hematopoietic stem cell dysfunction and dyshematopoiesis and thereby generalized dysplastic findings of bone marrow cells, as it is for the other bone marrow cells.

## References

[1] Olcay L, Yetgin S, Okur H, Erdemli E (2008). Rapid cell senescence and apoptosis in lymphocytes and granulocytes and absence of GM-CSF receptor in congenital dysgranulopoietic neutropenia. Leuk Res.

[2] Olcay L, Yetgin S, Erdemli E, Germeshausen M, Aktaş D, Büyükaşik Y, Okur H (2008). Congenital dysgranulopoietic neutropenia. Pediatr Blood Cancer.

[3] Dykstra MJ (1993.). A Manual of Applied Techniques for Biological Electron Microscopy. New York, Plenum Press.

[4] Olcay L, Yetgin S, Okur H, Erekul S, Tuncer M (2000). Dysplastic changes in idiopathic thrombocytopenic purpura and the effect of corticosteroids to increase dysplasia and cause hyperdiploid macropolycytes. Am J Hematol.

[5] Buyukasik Y, Karakus S, Goker H, Haznedaroglu IC, Ozatli D, Sayinalp N, Ozcebe OI, Dundar SV, Kirazli S (2002). Rational use of the PFA-100 device for screening of platelet function disorders and von Willebrand disease. Blood Coagul Fibrinolysis.

[6] Olcay L, Erdemli E, Kesimer M, Büyükasik Y, Okur H, Kalkanoğlu HS, Coskun T, Altay C (2005). High cystine in platelets from patients with nephropathic cystinosis: a chemical, ultrastructural, and functional evaluation. J Clin Pathol.

[7] Komarnichi M, Pietrzak I, Zozulinska M (1988). Mepacrine-labeled platelet dense-body number in patients with chronic uremia. Nephron.

[8] Pintado T, Maldonado JE (1976). Ultrastructure of platelet aggregation in refractory anemia and myelomonocytic leukemia. I. Ultrastructure of aggregation in normal controls and general defects in refractory anemia and myelomonocytic leukemia. Mayo Clin Proc.

[9] Pintado T, Maldonado JE (1976). Ultrastructure of platelet aggregation in refractory anemia and myelomonocytic leukemia. II. Individual platelet abnormalities: Thrombasthenia-like platelets, surface defects, and dissociation phenomena. Mayo Clin Proc.

[10] Poon IK, Lucas CD, Rossi AG, Ravichandran KS (2014). Apoptotic cell clearance: basic biology and therapeutic potential. Nat Rev Immunol.

[11] Olcay L, Ünal Ş, Onay H, Erdemli E, Öztürk A, Billur D, Metin A, Okur H, Yıldırmak Y, Büyükaşık Y, İkincioğulları A, Yılmaz-Falay M, Özet G, Yetgin S (1012). Granulocytic and non granulocytic lineages in children with congenital neutropenia and their non neutropenic parents: Biochemical, functional, morphological and genetic evaluation. Haematologica.

[12] Koval’chuk LV, Khoreva MV, Nikonova AS, Finogenova NA, Mamedova EA, Polovtseva TV, Fetisova LIa, Gracheva LA, Goldyreva NG (2010). Toll-like receptor-mediated functional activity of mononuclear cells in children with neutropenia. Zh Mikrobiol Epidemiol Immunobiol.

[13] Bohn G, Hardtke-Wolenski M, Zeidler C, Maecker B, Sauer M, Sykora KW, Grigull L, Welte K, Klein C (2008). Lethal graft-versus-host disease in congenital neutropenia caused by p14 deficiency after allogeneic bone marrow transplantation from an HLA-identical sibling. Pediatr Blood Cancer.

[14] Shitara T, Yugami S, Ijima H, Sotomatu M, Kuroume T (1994). Cytokine profile during high-dose rhG-CSF therapy in severe congenital neutropenia. Am J Hematol.

[15] Abbas AK, Lichtman AH, Pillai S (2015). Innate immunity. In: Cellular and Molecular Immunology. 8th ed. Canada, Elsevier Saunders,.

[16] Abbas AK, Lichtman AH, Pillai S (2015). Differentiation and functions of CD4+ effector T cells. In: Cellular and Molecular Immunology. 8th ed. Canada. Elsevier Saunders,.

[17] Geering B, Simon HU (2011). A novel signaling pathway in TNFα-induced neutrophil apoptosis. Cell Cycle.

[18] Ugan Y, Nazıroğlu M, Şahin M, Aykur M (2016). Anti-tumor necrosis factor alpha (Infliximab) attenuates apoptosis, oxidative stress, and calcium ion entry through modulation of cation channels in neutrophils of patients with ankylosing spondylitis. J Membr Biol.

[19] Bommhardt U, Chang KC, Swanson PE, Wagner TH, Tinsley KW, Karl IE, Hotchkiss RS (2004). Akt decreases lymphocyte apoptosis and improves survival in sepsis. J Immunol.

[20] Hotchkiss RS, Tinsley KW, Karl IE (2003). Role of apoptotic cell death in sepsis. Scand J Infect Dis.

[21] Kawakami Y, Tsukimoto M, Kuwabara K, Fujita T, Fujino O, Kojima S, Fukunaga Y (2011). Tumor necrosis factor-α-induced mononuclear cell death may contribute to polymorphonuclear cell predominance in the cerebrospinal fluid of patients with bacterial meningitis. J Nippon Med Sch.

[22] Cevik O, Adiguzel Z, Baykal AT, Somay G, Sener A (2013). The apoptotic actions of platelets in acute ischemic stroke. Mol Biol Rep.

[23] Kawada J, Kimura H, Shibata Y, Hara S, Hoshino Y, Kojima S, Nishikawa K, Morishima T (2006). Evaluation of apoptosis in Epstein-Barr virus-associated hemophagocytic lymphohistiocytosis. J Med Virol.

[24] McElhaney JE, Effros RB (2009). Immunosenescence: what does it mean to health outcomes in older adults?. Curr Opin Immunol.

[25] Freund A, Orjalo AV, Desprez PY, Campisi J (2010). Inflammatory networks during cellular senescence: Causes and consequences. Trends Mol Med.

[26] Olcay L, Billur D, Erdemli E, Baskin SE, Balci HF, Yetgin S (2015). Myelodysplastic features and cellular senescence in autoimmune disorders: a pilot study on patients with collagen tissue disorders and immune thrombocytopenic purpura. Turk J Med Sci.

[27] Thiele J, Lorenzen J, Manich B, Kvasnicka HM, Zirbes TK, Fischer R (1997). Apoptosis (programmed cell death) in idiopathic (primary) osteo-/myelofibrosis: naked nuclei in megakaryopoiesis reveal features of para-apoptosis. Acta Haematol.

[28] Olcay L, Tuncer AM, Okur H, Erdemli E, Uysal Z, Cetin M, Duru F, Cetinkaya DU (2009). Excessive naked megakaryocyte nuclei in myelodysplastic syndrome mimicking idiopathic thrombocytopenic purpura: a complicated pre- and post-transplantation course. Pediatr Hematol Oncol.

[29] Centurione L, Di Baldassarre A, Zingariello M, Bosco D, Gatta V, Rana RA, Langella V, Di Virgilio A, Vannucchi AM, Migliaccio AR (2004). Increased and pathologic emperipolesis of neutrophils within megakaryocytes associated with marrow fibrosis in GATA-1(low) mice. Blood.

[30] Aslan D, Yetgin S (2001). Megakaryocyte emperipolesis in a child with chronic neutropenia: an unusual coexistence. Turk J Pediatr.

[31] Houwerzijl EJ, Blom NR, van der Want JJ, Esselink MT, Koornstra JJ, Smit JW, Louwes H, Vellenga E, de Wolf JT (2004). Ultrastructural study shows morphologic features of apoptosis and para-apoptosis in megakaryocytes from patients with idiopathic thrombocytopenic purpura. Blood.

[32] Erber WN, Jacobs A, Oscier DG, O’hea AM, Mason DY (1987). Circulating micromegakaryocytes in myelodysplasia. J Clin Pathol.
